# Isolation and Characterization of Microsatellite Markers in Brown Planthopper (*Nilaparvata lugens* Stål)

**DOI:** 10.3390/ijms13089527

**Published:** 2012-07-27

**Authors:** Shengli Jing, Xi Zhou, Hangjin Yu, Bingfang Liu, Chunxiao Zhang, Shuzhen Wang, Xinxin Peng, Lili Zhu, Yi Ding, Guangcun He

**Affiliations:** State Key Laboratory of Hybrid Rice, College of Life Science, Wuhan University, Wuhan 430072, Hubei Province, China; E-Mails: jsl80@163.com (S.J.); cathychou@whu.edu.cn (X.Z.); yhj@whu.edu.cn (H.Y.); bingfangliu@163.com (B.L.); emilyat10@126.com (C.Z.); wangshuzhen04@163.com (S.W.); fred_pxx@126.com (X.P.); zhulili58@sina.com (L.Z.); yiding@whu.edu.cn (Y.D.)

**Keywords:** *Nilaparvata lugens*, microsatellites, polymorphism, genetic diversity

## Abstract

Brown planthopper (*Nilaparvata lugens* Stål) (Homoptera: Delphacidae) is an economically important pest on rice. In this study, 30 polymorphic microsatellite markers were developed from *N. lugens* genomic libraries using the method of Fast Isolation by AFLP of Sequence Containing Repeats (FIASCO). Polymorphism of each locus was detected in 48 individuals from two natural populations. These microsatellite loci revealed 2 to 18 alleles, and the expected and observed heterozygosities ranged from 0.042 to 0.937 and from 0.042 to 0.958, respectively. These markers will be useful for the future study of this agricultural pest in population genetics and molecular genetics.

## 1. Introduction

Brown planthopper (*Nilaparvata lugens* Stål) (Homoptera: Delphacidae) is a specialist insect pest of rice which does great damage to the rice plants not only directly by consuming the plants sap but also indirectly by transmitting rice viruses such as ragged stunt virus or grassy stunt virus. Many Asia rice-producing countries are frequently reported to have suffered significant damage and heavy yield losses due to brown planthopper [1]. Moreover, brown planthoppers are found to have adapted to the variety of host rice and formed new populations, which can break host plant’s resistance [2]. Therefore, it is important to study the population ecology and evolution of this pest. SSR, with advantages of multi-allelic behavior, co-dominance, abundance and high information content, have been developed for many species [3]. So far, the EST-SSR markers of brown planthopper have been developed for studying genetic diversity of the natural populations [4,5] and the experimental populations [6]. In general, genomic SSR markers are more polymorphic than EST-SSRs. In this study, we have developed and characterized another 30 genomic SSR markers for *N. lugens*.

## 2. Results and Discussion

A total of 136 primer pairs were designed from microsatellite sequences isolated from partial genome libraries by a standard Fast Isolation by AFLP of Sequence Containing Repeats (FIASCO) protocol. Out of them, sixty-two successfully yielded clear bands while the others showed multi-banding patterns or no amplification. Then, these primers were further tested in two natural populations of *N. lugens* collected from Wuhan, Hubei Province (Wuhan population) and Wuyishan, Fujian Province (Wuyishan population), respectively. Among them, 30 markers showed polymorphism between these two populations (Table 1); and the amplification products are within the expected size range.

It was found that the degree of polymorphism between two populations was not significantly different. The average number of detected alleles per locus and the mean observed heterozygosity for two populations were also similar. In the Wuhan population of *N. lugens*, the numbers of detected alleles per locus in 24 individuals ranged from 2 to 16, with an average of 9.2 alleles for the 30 markers. The expected and observed heterozygosities ranged from 0.042 to 0.910 (mean 0.717) and from 0.042 to 0.958 (mean 0.515), respectively (Table 2). The degree of polymorphism of BM1373 was the highest in the Wuhan population. Twenty-one loci (BM1362, BM1368, BM1369, BM1375, BM1377, BM1378, BM1392, BM1393, BM1417, BM1422, BM1423, BM1432, BM1433, BM1443, BM1446, BM1456, BM1462, BM1464, BM1472, BM1486 and BM1490) deviated significantly from HWE (*p* < 0.05) due to heterozygote deficiency, and null alleles were found in these loci except two (BM1362 and BM1486).

In the Wuyishan population, the numbers of detected alleles per locus in 24 individuals ranged from 2 to 18, with an average of 9.3 alleles per locus. The expected and observed heterozygosities ranged from 0.191 to 0.937 (mean 0.717) and from 0.042 to 0.875 (mean 0.530), respectively. The degree of polymorphism of BM1373 was also the highest in the Wuyishan population. Eighteen loci (BM1368, BM1369, BM1372, BM1375, BM1378, BM1392, BM1393, BM1417, BM1418, BM1422, BM1422, BM1432, BM1433, BM1446, BM1456, BM1471, BM1472 and BM1490) deviated significantly from HWE (*p* < 0.05) due to heterozygote deficiency, and null alleles were found in these loci except three (BM1417, BM1418 and BM1456).

The statistical significance of the linkage disequilibrium among 30 microsatellite loci was tested by Fisher’s exact probability test. Linkage disequilibrium *p*-values were obtained for 435 pairs of marker combinations. Out of these, 79 (18.2%) pairs in the Wuhan population and 91 (20.9%) pairs in the Wuyishan population, showed significant LD at *p* < 0.05, respectively.

Genomic SSR markers appear to be more polymorphic in this study. Both the number of alleles and the observed heterozygosity of genomic SSR dataset are higher than those of two EST-SSR datasets [5,6]. In Liu and Hou’s study, the number of alleles ranged from two to five, and the observed heterozygosity ranged from 0.111 to 0.411. In Jing’s study, the number of alleles ranged from two to seven, and the average observed heterozygosity for four populations ranged from 0.43 to 0.52. In this study, these microsatellite loci revealed 2 to 18 alleles, and the observed heterozygosities ranged from 0.042 to 0.958. Therefore, these microsatellite loci are better than EST-SSRs for genetic diversity study and the construction of linkage map of *N. lugens*.

The observed heterozygosity was lower than the expected heterozygosity in all loci except seven (BM1415, BM1420, BM1476 and BM1483 in both populations; BM1372, BM1373 and BM147 in the Wuyishan population). Several factors may lead to the observed heterozygosity being less than expected heterozygosity in a population, such as the presence of null alleles and sex-linkage that are two aspects of great importance for explaining the disequilibrium of HW. In this study, the null alleles were present in many loci, while the evidence of sex was not found because only female adults were used. Therefore, further investigation for these two factors is needed in future studies, especially for the sex-like loci.

## 3. Experimental Section

The genomic DNA was extracted from a female individual of *N. lugens* with a CTAB protocol [7]. The (AC)_13_ and (AAG)_8_-enriched partial genomic libraries were constructed, employing a AFLP (amplified fragment length polymorphism) of sequences containing repeats (FIASCO) protocol [8]. Fragments containing microsatellite repeats were cloned into pUC18-T vector (TaKaRa) and transformed into TOP10 cells. Finally, 219 positive clones with suitable insert length were identified and sequenced using an ABI 3730 DNA sequencer.

189 sequences were obtained and screened for the SSR motifs using the SSRIT discover program [9]. As a result, 87 sequences contained at least one microsatellite locus, and 136 primer pairs were designed by using BatchPrimer3 [10]. For all PCR amplifications, we used a PTC-100 thermal cycler (MJ Research) and performed in 10 μL volumes containing 10 ng of template DNA, 0.3 μM of each of the two primers, 0.2 mM deoxynucleotide triphosphates (dNTPs), 2.5 mM MgCl_2_, 1 × PCR buffer, and 1 unit of *Taq* DNA polymerase (Fermentas). The PCR cycling program, in each case, was 94 °C for 5 min, followed by 35 cycles of 94 °C for 15 s, 55 °C for 15 s, and 72 °C for 30 s, with a final extension step of 72 °C for 10 min. PCR amplification products were size-fractionated by electrophoresis on 6% denaturing polyacrylamide sequencing gels that were run at a constant power of 60 W, and then detected by silver staining [11]. Allele sizes were scored by comparison with pBR322 DNA/*Msp*I DNA size markers (Tiangen Biotech).

The level of polymorphism was determined for 48 female adults from two populations of *N. lugens* collected from rice fields in Wuhan, Hubei Province and in Wuyishan, Fujian Province, China. For each locus, the number of alleles (*N*_a_), observed heterozygosity (*H*_o_), expected heterozygosity (*H*_e_), tests for linkage disequilibrium (LD) and deviations from Hardy-Weinberg equilibrium (HWE) were calculated by the software Arlequin 3.1 [12]. The occurrence of a null allele was estimated by the software MICRO-CHECKER [13].

## 4. Conclusions

In summary, 30 microsatellite markers have been developed from *Nilaparvata lugens*, and reveal a high degree of polymorphism among individuals in two natural populations. These markers are useful for population genetic diversity and molecular genetics study of this agricultural pest.

## Figures and Tables

**Table 1:** Characteristics of 30 new microsatellite markers developed in *Nilaparvata lugens.*

Locus	Repeat motif	Primer sequence (5′–3′)	GenBank accession No.
BM1362	(AAGT)3	**F:** TGGGCAAGACCATCTTGATA **R:** CGAATTCAAATGGGAAGTCTGT	JQ967334
BM1368	(AG)10	**F:** AGGGATTCAGATTCAGGGAGA **R:** CGGTGGAGATGAAAGTGGAC	JQ967337
BM1369	(AG)9	**F:** TCGTGCAGAAGCGAAAGAAT **R:** TTTTTCAACTCGTCAGCATGT	JQ967338
BM1372	(AT)6	**F:** GTGCAACCACGAGCATTTC **R:** AAGACCCCTTCCTCAGCATC	JQ967341
BM1373	(CT)7	**F:** CCACATTCCACCTCTTTTCA **R:** AGTGCGCAGAAACTTGATGA	JQ967340
BM1375	(GA)8	**F:** TCCATGAGAAAGAGGGCTTG **R:** GCTGAGGCCTTACCTATCAAA	JQ967346
BM1377	(CTT)4	**F:** TACACTAACGCACGCACACA **R:** TCAACGGTAAAGGGAGAAGG	JQ967347
BM1378	(TG)7	**F:** CATCATTGCAACGTTCATCC **R:** GCCCTCCAAATTAGGTCTCC	JQ967348
BM1392	(AAG)4	**F:** GAAGCTGAAAAAGAAAGATGAAGAA **R:** TTTGCCTCAATTTTGCTCCT	JQ967349
BM1393	(CCT)5	**F:** ACCTCTCCCCCTCATCATTC **R:** TGGTTTGGTGTTCGATGCTA	JQ967350
BM1415	(TC)6	**F:** CGGTCCAAAAATGGAAAATG **R:** GGGTGTGTGCCATGATTTAG	JQ967360
BM1417	(TGAA)3	**F:** TGAGTTGGAAGGTGTCATGG **R:** TCCTCAATGGACCTCTCTCCT	JQ967353
BM1418	(ATG)5	**F:** GAAAGAAAATGGAGCCGTCA **R:** ACCCATGCCTCTTTCCTCTT	JQ967354
BM1420	(GAAG)3	**F:** GAAACTTGGTGAGGGGATCA **R:** TTCTTTGTTCACAATTTTCTCAGC	JQ967342
BM1422	(GA)6	**F:** TAAGGCGAGAAAGTGCGATT **R:** CTTTCTCCCACTTCCCCATC	JQ967343
BM1423	(GAT)6	**F:** GGAGGAGGTCGAGGAAGAAT **R:** TCCTCCATTCCTTCTTCTTGTT	JQ967344
BM1432	(GA)7	**F:** GTGACAAAGAGCGAGGGAGT **R:** CGCCCTAACTTACCCTGCTA	JQ967335
BM1433	(AG)8	**F:** TGCAGAGAGATGAGGCAAAA **R:** TTTCGCACAACGTACTGCTC	JQ967336
BM1437	(TCAA)3	**F:** CAAACAATAGCGAGCATTACAGA **R:** CCAGCGTTATTGTCCTGTCA	JQ967339
BM1443	(GATT)4	**F:** TCCTTCCCATCAATACAAGACC **R:** TCAAGCCCTCTTTCTCATGG	JQ967346
BM1446	(TC)11	**F:** TTTGTCGGAGCGATCTCTTT **R:** CGCTGTCCATTCAACAAATG	JQ967345
BM1456	(TAA)5	**F:** TGGAAGTGAAACTGCAAGAAAA **R:** TTGCGACCTGAAAACTCTGA	JQ967357
BM1462	(AAG)12	**F:** GTCCGGGCTTAGCCTTTTAT **R:** GCATCTAACGGGTGATTCTCA	JQ967351
BM1464	(AG)7	**F:** CATTCACAGCTGAGGTATGAGG **R:** CACAGCTTGACTCACCCTCTC	JQ967359
BM1471	(AAG)4	**F:** CGAAGCGGAAATAGATGGTT **R:** CACATTTTCCAGGCTTCACC	JQ967355
BM1472	(GAA)7	**F:** GGGAAGGGGAGAAGTCAAAG **R:** CATTCCACCTCCTTCTTCCA	JQ967358
BM1476	(GAAGGA)4	**F:** CGACGGAAAATCAGTCATCA **R:** CCTGCTTCACATCCTCCTTC	JQ967356
BM1483	(AAT)4	**F:** GCGTTTGAGCGTGGTTTCTA **R:** ATGGAGTGGGTCCACCAATA	JQ967352
BM1486	(AAG)5	**F:** AAAAATGGATGGGAAAGGAGA **R:** CCTTCCATCCTTTTATTCTTCTCA	JQ967354
BM1490	(GT)11	**F:** GTCAAATCCCTGGCACATTT **R:** TGAAGTGAATGAAACCCACATC	JQ967344

**Table 2:** Diversity estimation in two populations of *Nilaparvata lugens.*

Locus	Population WH (*n* = 24)					Population WYS (*n* = 24)				
	*N*_a_	*H*_o_	*H*_e_	*D*	*S*	*N*_a_	*H*_o_	*H*_e_	*D*	*S*
BM1362	4	0.292	0.393	*	232–246	5	0.292	0.425	NS	232–252
BM1368	9	0.391	0.844	*	130–162	10	0.292	0.544	*	138–168
BM1369	11	0.667	0.877	*	182–208	9	0.409	0.819	*	186–308
BM1372	2	0.042	0.042	NS	200–204	2	0.042	0.191	*	200–204
BM1373	15	0.958	0.902	NS	110–152	16	0.875	0.928	NS	116–172
BM1375	11	0.583	0.853	*	180–206	12	0.625	0.886	*	180–238
BM1377	9	0.458	0.732	*	194–224	6	0.542	0.722	NS	187–232
BM1378	12	0.375	0.883	*	192–240	18	0.409	0.937	*	168–240
BM1392	13	0.542	0.766	*	148–280	15	0.545	0.814	*	130–250
BM1393	5	0.250	0.527	*	186–246	10	0.375	0.707	*	186–246
BM1415	7	0.708	0.701	NS	192–212	7	0.542	0.448	NS	180–216
BM1417	3	0.273	0.588	*	180–200	4	0.458	0.621	*	180–204
BM1418	9	0.583	0.689	NS	164–194	8	0.542	0.743	*	164–194
BM1420	6	0.292	0.270	NS	212–260	4	0.478	0.467	NS	200–260
BM1422	10	0.500	0.853	*	182–206	13	0.565	0.921	*	178–204
BM1423	13	0.652	0.909	*	217–320	13	0.636	0.874	*	220–379
BM1432	15	0.739	0.910	*	130–218	12	0.261	0.875	*	140–218
BM1433	12	0.565	0.898	*	184–250	13	0.591	0.906	*	186–244
BM1437	7	0.500	0.505	NS	182–230	6	0.565	0.593	NS	174–230
BM1443	11	0.348	0.750	*	168–240	7	0.542	0.588	NS	160–202
BM1446	16	0.652	0.907	*	164–254	13	0.609	0.891	*	170–254
BM1456	10	0.458	0.828	*	140–220	5	0.500	0.582	*	180–260
BM1462	12	0.739	0.901	*	179–260	11	0.792	0.855	NS	179–216
BM1464	5	0.500	0.739	*	182–280	4	0.556	0.743	NS	182–280
BM1471	4	0.682	0.601	NS	201–252	4	0.300	0.750	*	198–252
BM1472	12	0.333	0.895	*	161–238	15	0.565	0.919	*	138–260
BM1476	6	0.500	0.429	NS	180–222	8	0.818	0.648	NS	180–240
BM1483	8	0.708	0.679	NS	204–243	10	0.810	0.724	NS	207–261
BM1486	6	0.583	0.736	*	190–217	7	0.667	0.707	NS	190–262
BM1490	14	0.583	0.902	*	184–238	12	0.708	0.887	*	176–210
Mean	9.2	0.515	0.717	-	-	9.3	0.530	0.724	-	-

*N*: population sample size; *H*_O_: observed heterozygosity; *H*_e_: expected heterozygosity; *N*_a_: number of alleles; *D*: deviation from Hardy-Weinberg equilibrium; *S*: Size range (bp); NS: not significant;

* significant deviations from Hardy-Weinberg expectations (*p* < 0.05); Population WH: Wuhan population; Population WYS: Wuyishan population.
